# Paraoxonase-2 contributes to promoting lipid metabolism and mitochondrial function via autophagy activation

**DOI:** 10.1038/s41598-022-25802-1

**Published:** 2022-12-12

**Authors:** Gu-Choul Shin, Hyeong Min Lee, Nayeon Kim, Sang-Ku Yoo, Hyung Soon Park, Leo Sungwong Choi, Kwang Pyo Kim, Ah-Ra Lee, Sang-Uk Seo, Kyun-Hwan Kim

**Affiliations:** 1 Department of Precision Medicine, School of Medicine, Sungkyunkwan University, Suwon, 16419 Republic of Korea; 2 Department of Microbiology College of Medicine, Songeui Medical Campus, The Catholic University of Korea, Seoul, 06591 Republic of Korea; 3 Department of Applied Chemistry, Institute of Natural Science, Global Center for Pharmaceutical Ingredient Materials, Kyung Hee University, Yongin, 446-701 Republic of Korea; 4 Department of Biomedical Science and Technology, Kyung Hee Medical Science Research Institute, Kyung Hee University, Seoul, 02453 Republic of Korea; 5 Glaceum Inc., Suwon, Republic of Korea

**Keywords:** Cell biology, Molecular biology

## Abstract

Non-alcoholic fatty liver disease (NAFLD) is an increasingly prevalent immuno-metabolic disease that can progress to hepatic cirrhosis and cancer. NAFLD pathogenesis is extremely complex and is characterized by oxidative stress, impaired mitochondrial function and lipid metabolism, and cellular inflammation. Thus, in-depth research on its underlying mechanisms and subsequent investigation into a potential drug target that has overarching effects on these features will help in the discovery of effective treatments for NAFLD. Our study examines the role of endogenous paraoxonase-2 (PON2), a membrane protein with reported antioxidant activity, in an in vitro cell model of NAFLD. We found that the hepatic loss of PON2 activity aggravated steatosis and oxidative stress under lipotoxic conditions, and our transcriptome analysis revealed that the loss of PON2 disrupts the activation of numerous functional pathways closely related to NAFLD pathogenesis, including mitochondrial respiratory capacity, lipid metabolism, and hepatic fibrosis and inflammation. We found that PON2 promoted the activation of the autophagy pathway, specifically the mitophagy cargo sequestration, which could potentially aid PON2 in alleviating oxidative stress, mitochondrial dysfunction, lipid accumulation, and inflammation. These results provide a mechanistic foundation for the prospect of PON2 as a drug target, leading to the development of novel therapeutics for NAFLD.

## Introduction

Non-alcoholic fatty liver disease (NAFLD) is a progressive chronic liver disease that may result in steatohepatitis, fatty liver fibrosis, liver cirrhosis, and hepatocellular carcinoma (HCC)^1^. The occurrence of NAFLD is associated with major abnormalities in hepatic lipid metabolism^1^. Imbalance of lipid metabolism leads to abnormal cellular lipid composition, accumulation of toxic lipid metabolites, and increased oxidative stress in hepatocytes, which eventually leads to mitochondrial damage, cell death, and inflammation^2,3^. During liver inflammation, damaged liver cells are replaced by an observed proliferation of hepatocytes and an increase in the gene expression of extracellular matrix (ECM) proteins^4,5^. However, the excessive deposition of ECM leads to liver fibrosis, which can be fatal.

Fundamentally, mitochondria are the primary organelles for lipid and glucose metabolism; therefore, they are also major sources of reactive oxygen species (ROS), which are normally cleared by endogenous mitochondrial antioxidants^6^. However, in the liver cells of patient with NAFLD, the imbalance between excessive ROS and reduced mitochondrial antioxidant capacity leads to increased lipid peroxidation and subsequent mitochondrial damage^7^. As an adaptive mechanism, autophagy kicks in to clear any damaged mitochondria, thus catalyzing the initiation of mitochondrial biogenesis. Hence, autophagy plays an important role in mitochondrial homeostasis^8^. Autophagy also contributes to lipid metabolism homeostasis via degradation of lipid droplets (LDs)^9^. Hence, the efficiency of the autophagy pathway is crucial for protecting liver cells, alleviating liver inflammation, and eventually resolving NAFLD^8,10,11^. Therefore, key regulators of these pathways have been proposed as potential targets for the development of drug candidates to effectively treat NAFLD.

Paraoxonase-2 (PON2) is the oldest member of the PON family, consisting of extracellular, plasma-associated PON1 and PON3 and intracellular PON2. The intracellular localization of PON2 has made it harder to study when compared to PON1^12^. PON2, like PON1, is an enzyme with undefined antioxidant properties, and PON2 deficiency is closely associated with the development of atherosclerosis in a mouse model^13^. The loss of PON2 activity led to mitochondrial dysfunction, accompanied with increased mitochondrial oxidative stress in macrophages, and increased levels of oxidized low-density lipoprotein (LDL) in serum, which is associated with increased inflammation and atherogenic features. In addition to PON2 and its anti-atherogenic effects, another study showed that an increase in oxidative stress in the liver of rats with NAFLD is also accompanied by decreased hepatic PON2 activity^14^, suggesting that impaired PON2 activity in liver cells may be closely associated with the development and progression of NAFLD. However, the exact mechanism via which PON2 preserves hepatic homeostasis and metabolism under lipotoxic stress is unknown.

In this study, we investigated the potential role of PON2 in an in vitro cell model of NAFLD. We found that PON2 is crucial for preserving metabolic homeostasis in hepatocytes by increasing autophagy activity, as well as antioxidant capacity, both of which lead to an alleviation of lipid accumulation, a decrease in ECM deposition, reduction of oxidized lipid metabolites, and mitigation of inflammation. These findings illustrate the prospect of PON2 as a potential target for the development of drugs to effectively treat patients with immunometabolic diseases like NAFLD.

## Results

### Hepatic PON2 is inactivated by lipid-mediated oxidative stress

**Figure 1:**
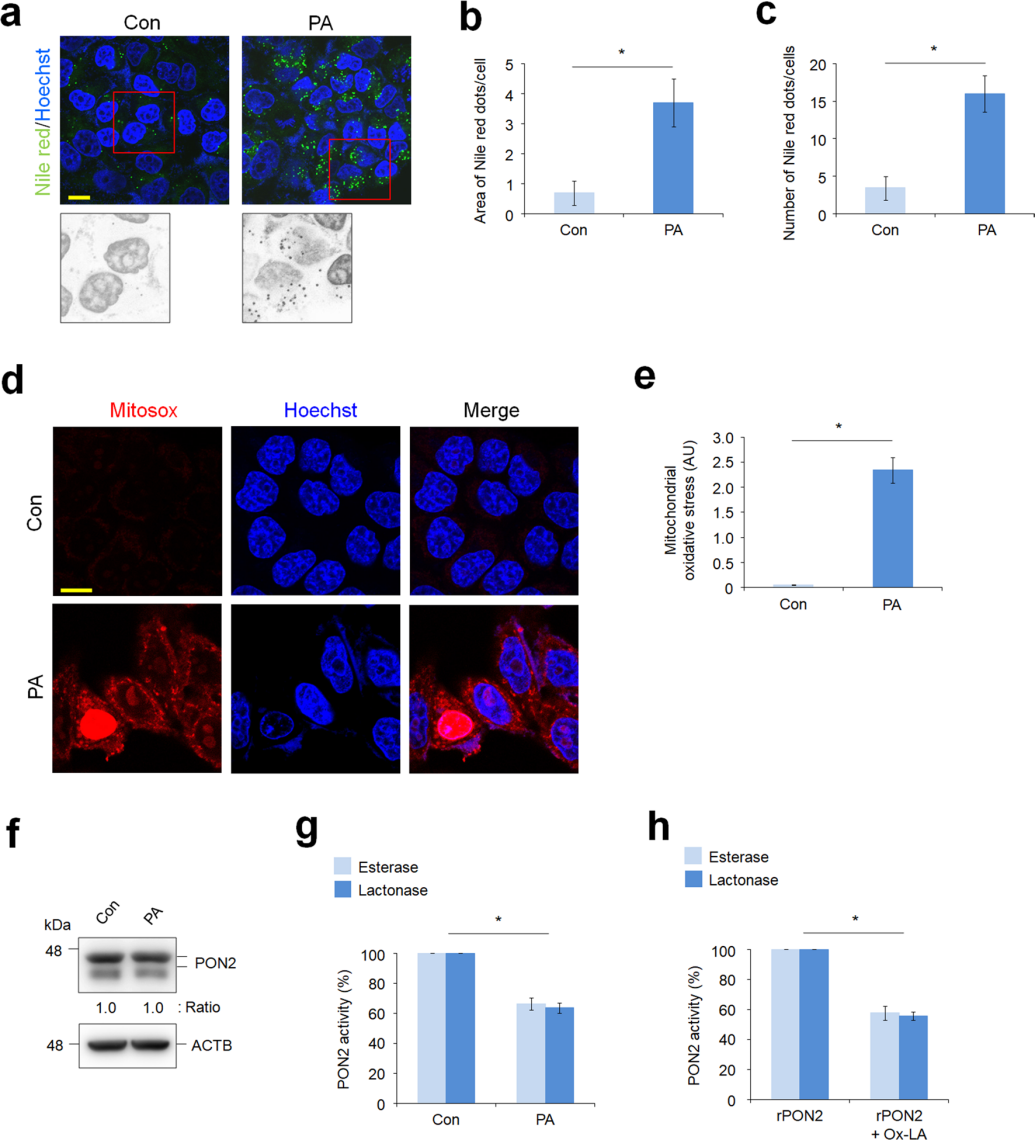
Palmitic acid (PA)-mediated lipotoxic condition inhibits the paraoxonase-2 (PON2) activity in hepatocytes. (**a**) Confocal fluorescence analysis showing PA-induced lipid accumulation. L02 hepatocytes were treated with PA (100 μM) for 24 h and stained with Nile red to determine lipid accumulation. Representative images from three independent experiments are shown (scale bar = 50 μm). (**b**,**c**) Quantification of Nile red staining intensities is measured by the area of Nile red dots per cells (**b**) and by the number of Nile red dots per cells (**c**). (**d**) Confocal fluorescence images showing the generation of mitochondrial superoxide, which was analyzed using MitoSox staining. L02 cells were treated with PA for 24 h and stained with MitoSox. Cell nuclei were stained with Hoechst 33342. Representative images from three independent experiments are shown (scale bar = 50 μm). (**e**) Bar plots of the quantification of MitoSox staining intensities are shown. (**f**) Immunoblot analysis of PON2 expression in cells treated with or without PA. ACTB was used as a loading control. Band intensities of the indicated proteins are shown below. (**g**) Endogenous PON2 activities in L02 cells treated with or without PA for 24 h. Bar plots of the average esterase and lactonase activities are shown (n = 3). (**h**) Purified recombinant PON2 activities incubated with or without oxidized linoleic acid (Ox-LA) for 10 min. Bar plots of the average esterase and lactonase activities of recombinant PON2 (n = 3). Error bars indicate standard deviation. Statistical significance between two groups was determined with two-tailed Student’s *t* test. *p < 0.05.

In patients with NAFLD, palmitic acid (PA) is one of the most abundant saturated FAs in plasma and plays a dominant role in generating lipotoxicity in hepatocytes^15,16^. To investigate whether hepatic steatosis affects PON2 expression and activity, we used in vitro fatty liver model that induces lipid accumulation in L02 normal liver cells via PA treatment. Nile red staining showed significant LD accumulation in L02 cells treated for 24 h with PA (Fig. 1a), whereas minimal staining for lipids was seen in control cells that were not treated with PA. Analysis of Nile red dots and numbers indicated that accumulation of LDs was induced in almost all PA-treated cells (Fig. 1b,c). PA is used as an energy source for FA oxidation and TCA cycle processes in mitochondria. However, PA catabolic processes also induce mitochondrial ROS production, leading to lipotoxicity in hepatocytes through the accumulation of mitochondrial ROS^17^. To examine this phenomenon, we measured mitochondrial superoxide production in PA-treated cells. As expected, the intensity of Mitosox, a mitochondrial superoxide indicator, was remarkably increased by PA treatment in L02 cells compared to that in control cells (Fig. 1d,e). Under lipo-oxidative stress condition, we hypothesized that lipid peroxidation and toxic lipid metabolites would be accumulated in cells, leading to potential changes in the activity of various metabolic enzymes. Thus, we investigated whether PA-induced oxidative stress can affect the expression and activity of PON2. Evidently, PA treatment did not affect the expression levels of PON2 (Fig. 1f) but decreased both the esterase and lactonase activity of PON2 (Fig. 1g). To validate this effect, we measured activity of PON2 in a cell-free system using purified PON2 protein and Ox-LA. Remarkably, Ox-LA treatment inhibited both esterase and lactonase activity of purified PON2 enzyme, similar to the PON2 activity inhibition observed in PA treatment (Fig. 1h). These results indicate that PON2 activity is easily influenced by oxidative stress under lipotoxic conditions, as seen in NAFLD.

### PON2 knock-down disrupts various interdependent pathways involved in NAFLD pathogenesis

**Figure 2:**
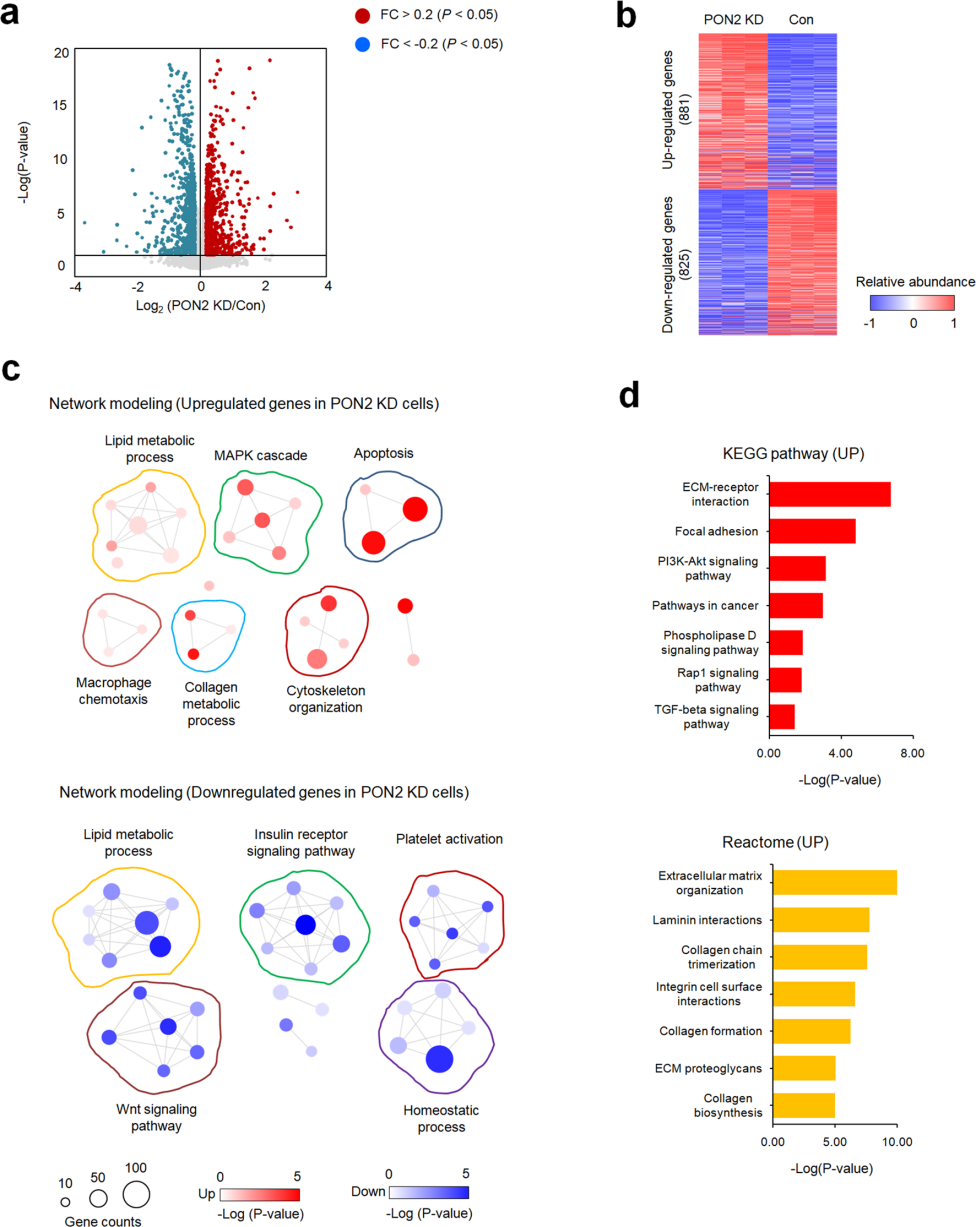
Transcriptome analyses using RNA-seq data in PON2-deficient and control cells. (**a**) Volcano plot of hepatic differentially expressed genes (DEGs) between the PON2 depletion and control. Genes upregulated or downregulated by more than 1.2-fold are shown in red and blue, respectively. (**b**) Two-dimensional hierarchical clustering of DEGs between different pairs of PON2-deficient and control cells. In total, 881 and 825 genes are upregulated and downregulated by PON2 depletion compared to that in control, respectively. (**c**) Network modeling scheme showing the most significant Gene Ontology (GO) biological processes for each cluster of genes upregulated (upper panel) and downregulated (bottom panel) in PON2-deficient cells relative to that in the control cells. Red and blue denote highly and weakly expressed genes, respectively, and circle size indicates gene counters. The legends for color-coding and gene counting are shown below. (**d**) Top-ranked pathways that are significantly altered in PON2-deficient cells relative to those in control cells. Upper panel represent the KEGG pathways enriched by genes exhibiting upregulated expression in PON2-deficient cells relative to those in control cells. Bottom panel represent the Reactome pathways enriched by the genes exhibiting upregulated expression in PON2-deficient cells relative to those in control cells. Each cell is color-coded based on the fold change in the expression of genes in PON2-deficient cells, relative to control cells.

To explore the potential functions of PON2 in hepatocytes, we generated a PON2-KD liver cell line and performed RNA-seq analysis to determine DEGs between PON2-KD and control cells. We detected 825 and 881 genes that exhibited more than 1.2-fold decrease or increase in mRNA expression, respectively (Fig. 2a,b). Gene network modeling analysis on these gene sets revealed that the expression of genes involved in lipid metabolic processes, macrophage chemotaxis, collagen metabolic processes, cytoskeleton organization, apoptosis, and MAPK cascade was increased in PON2-KD hepatocytes (Fig. 2c, Supplementary Fig. 1a). By contrast, the expression of genes associated with FA synthesis, insulin receptor signaling pathway, platelet inactivation, homeostatic processes, and Wnt signaling pathway was decreased in PON2-KD hepatocytes (Fig. 2c, Supplementary Fig. 1b). To improve the data resolution, the DEGs in PON2-KD cells were further analyzed using the KEGG and Reactome pathway database (Fig. 2d). Notably, the upregulated gene set was highly enriched in pathways responsible for inflammatory responses and liver fibrosis, ECM remodeling, and cell adhesion, whereas the downregulated gene set was not significantly enriched for any functional pathways. These results underline a potentially important role of hepatic PON2 in driving NAFLD pathogenesis.

### PON2 depletion impairs hepatic lipid metabolism, leading to intracellular lipid accumulation

**Figure 3:**
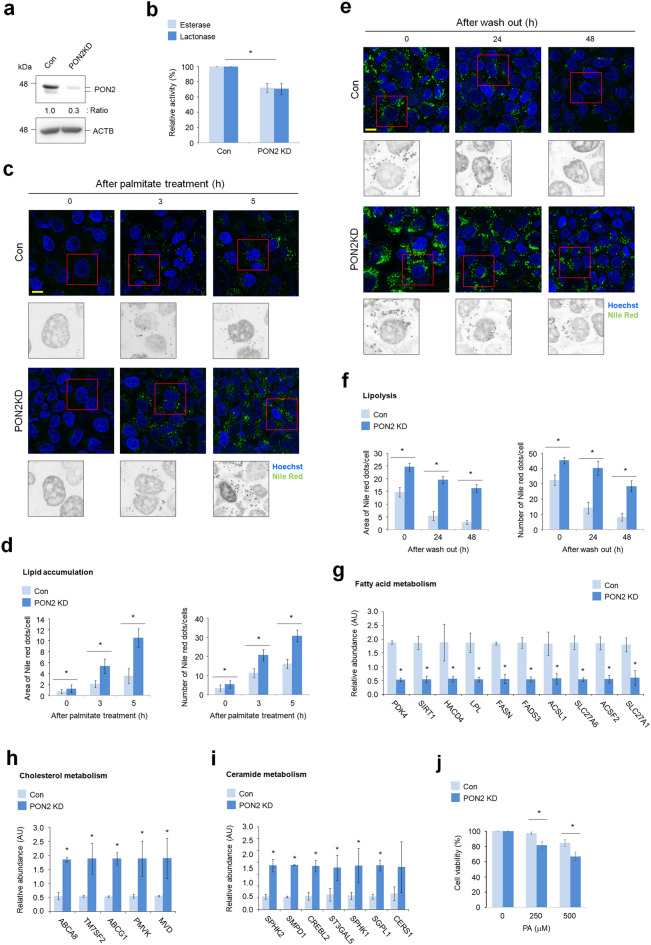
PON2 promotes lipid catabolic pathways in hepatocytes. (**a**) Immunoblot analysis of PON2 expression in PON2-deficient and control cells. ACTB was used as a loading control. Band intensities of the indicated proteins are shown below. (**b**) Endogenous PON2 activities in the PON2-deficient and control L02 cells. Bar plots of the average esterase and lactonase activities are shown (n = 3). (**c**) Confocal fluorescence analysis showing time-dependent lipid accumulation after PA treatment in PON2-deficient and control cells. Cells were stained with Nile red at indicated time after PA treatment (n = 3; scale bar = 50 μm). (**d**) Quantification of Nile red staining intensities is measured by the area of Nile red dots per cells and by the number of Nile red dots per cells. (**e**) Confocal fluorescence analysis showing lipolysis activity in PON2-deficient and control cells. Cells were treated with PA for 24 h, replenished with normal growth media, and then stained with Nile red at the indicated time after wash out of PA. Representative images from three independent experiments are shown (scale bar = 50 μm). (**f**) Quantification of Nile red staining intensities is measured by the area of Nile red dots per cells and by the number of Nile red dots per cells. (**g**) The expression of genes related to FA metabolism in PON2-deficient cells relative to that in control cells. (**h**) The expression of genes related to cholesterol metabolism in PON2-deficient cells relative to that in controls. Bar plots of the average expression of genes are shown. (**i**) The expression of genes related to ceramide metabolism in PON2-deficient cells relative to that in controls. (**j**) PA-mediated lipotoxicity in PON2-deficient cells compared to the control cells. Error bars indicate standard deviation. Statistical significance between two groups was determined with two-tailed Student’s *t* test. *p < 0.05.

Following the transcriptome profiling analyzed in PON2-deficient liver cells, we investigated the potential role of PON2 in regulating the lipid metabolism. In PON2-deficient liver cells, as verified by using western blotting and its enzyme activity assay (Fig. 3a,b), PA treatment increased accumulation of LDs compared to that in control cells (Fig. 3c,d). We then investigated the role of PON2 on lipolysis activity of hepatocytes. Accumulated LDs were significantly eliminated after 48 h of PA treatment in control cells; however, such elimination was not observed in PA-treated PON2-KD cells (Fig. 3e,f). These results demonstrate that PON2 has a phenotype-altering role in effective regulation of hepatic lipid catabolism. To investigate this further, we analyzed the mRNA expression of key genes related to lipid metabolism. We found that the expression levels of various genes related to cholesterol and ceramide biosynthesis were relatively higher in PON2-deficient cells than in control cells (Fig. 3g,h). By contrast, the expression of key genes related to FA metabolism was decreased by PON2 depletion (Fig. 3i). These data indicate that many key genes related to lipid metabolism were influenced by PON2 depletion, suggesting that PON2 function is possibly associated with lipid homeostasis in hepatocytes. To investigate the potential function of PON2 in hepatocyte lipotoxicity, we determined the cell viability of PON2-deficient cells under PA treatment. The PA-mediated cytotoxicity was significantly higher in a dose-dependent manner in PON2-KD cells than in control cells (Fig. 3j). This result indicates that potential role of PON2 is important for the protection of hepatocyte under lipotoxic condition.

### PON2 depletion leads to mitochondrial dysfunction and aggravates oxidative stress upon PA treatment of hepatocytes

**Figure 4:**
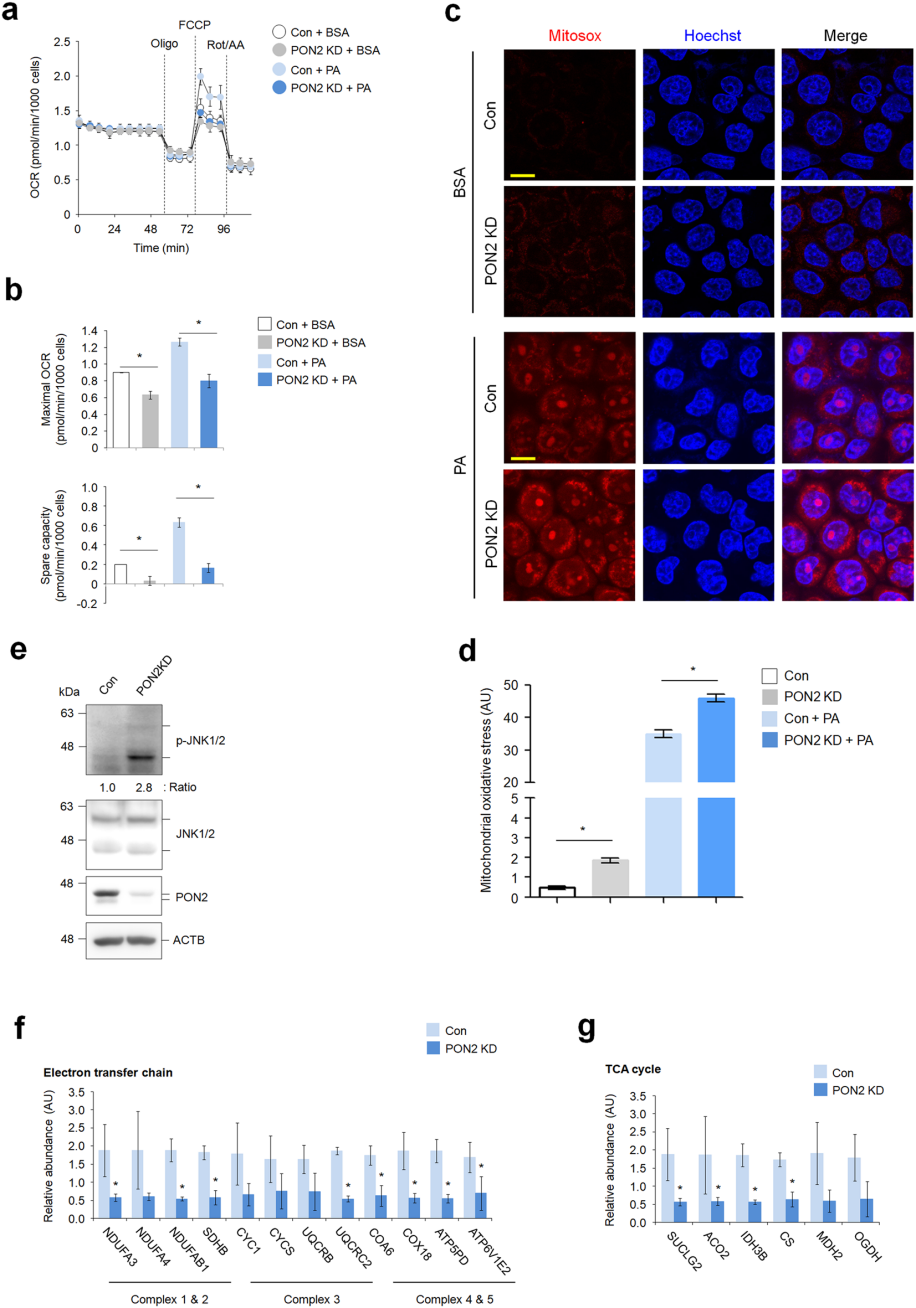
PON2 depletion impairs mitochondrial metabolism and oxidative stress. (**a**) Seahorse measurement of oxygen consumption rate (OCR) in PON2-deficient and control cells treated with or without PA. (**b**) Bar plots of maximal OCR and spare respiratory capacity are shown. Data were obtained from three independent experiments. (**c**) Confocal fluorescence images showing the generation of mitochondrial superoxide, which was analyzed using MitoSox. PON2-deficient and control cells were treated with or without PA for 24 h and stained with MitoSox. Cell nuclei were stained with Hoechst 33342. Representative images from three independent experiments are shown above (scale bar = 50 μm). (**d**) Bar plots of the MitoSox staining intensity are shown below. (**e**) Immunoblotting analysis of phosphorylation of JNK1/2 as marker for mitochondrial oxidative stress in PON2-deficient and control cells. ACTB was used as a loading control. Band intensities of indicated proteins are shown below. (**f**) The expression of genes related to mitochondrial electron transfer chain in PON2-deficient cells relative to controls. Bar plots of the average expression of genes are shown. (**g**) The expression of genes related to mitochondrial TCA cycle in PON2-deficient cells relative to controls.

To determine whether PON2 depletion affects mitochondrial respiration, we measured oxygen consumption rate (OCR using the Seahorse XF kits and obtained an OCR profile plot (Fig. 4a). Subsequently, we analyzed six different bioenergetic measurements, including non-mitochondrial respiration, basal respiration, proton leak, maximal respiration, ATP-linked respiration, and spare respiratory capacity, all to estimate mitochondrial function. Under basal conditions, the maximal OCR and spare respiratory capacity in PON2-KD cells were shown to be lower than those in control cells. Similarly, PON2 depletion also resulted in a lower maximal OCR and spare respiratory capacity than those in control cells upon PA treatment (Fig. 4b). However, the basal OCR, ATP-linked respiration, proton leak, and non-mitochondrial respiration during FA oxidation were not shown to be significantly different between PON2-deficient and control cells (Supplementary Fig. 2). Decrease in maximal respiration is often an indicator of mitochondrial dysfunction, including impaired mitochondrial biogenesis, whereas spare respiratory capacity is an indicator of the cellular ability to respond to stress and energy demand. Thus, our data strongly indicate that PON2 is involved in maintaining mitochondrial respiratory capacity, especially related to FA catabolism upon excessive lipid accumulation.

Increased mitochondrial FA oxidation has been proposed as the main process that leads to ROS generation in the event of excess lipid accumulation^18^. Notably, the oxidation of PA causes increased generation of superoxide in several cell types, including hepatocytes^19^. However, the molecular mechanisms behind cellular ROS generation by PA remain to be fully elucidated. To assess the potential role of PON2 on ROS generation by PA, we measured superoxide generation using the superoxide-specific indicator MitoSox. No fluorescence was detected in untreated control cells, whereas marginal fluorescence signal was shown in untreated PON2-deficient cells (Fig. 4c,d). When control cells were treated with PA, there was an intense fluorescence signal of Mitosox. Furthermore, PON2-deficient cells treated with PA showed higher fluorescence intensity than that in treated control cells. These results point towards the potential role of PON2 as a mitochondrial antioxidant, both under basal and lipotoxic conditions.

Mitochondrial ROS accumulation activates the JNKs, leading to mitochondrial dysfunction^19,20^. Using this information, we assessed whether PON2 depletion induces JNK activation to confirm the role of PON2 as antioxidant factor. Immunoblot analysis using antibody for phosphorylated JNKs showed an increased activation of JNKs in PON2-KD cells compared to that in control cells (Fig. 4e). Our data indicated that PON2 depletion induced oxidative stress-mediated JNK activation, which is known to be involved in mitochondrial dysfunction. In addition, we investigated the mRNA expression of genes that regulate mitochondrial metabolism. For instance, we found that the expression of various genes related to regulation of the electron transfer chain (ETC) and TCA cycle was decreased in PON2-deficient cells compared to that in control cells (Fig. 4f,g). Collectively, our data indicate that PON2 has dual functions in maintaining of mitochondrial homeostasis—first, via promotion of mitochondrial metabolic activity; second, by protection of oxidative stress-mediated mitochondrial damage in hepatocytes.

### PON2 depletion increases oxidized lipid metabolites and inflammatory response in hepatocytes

**Figure 5:**
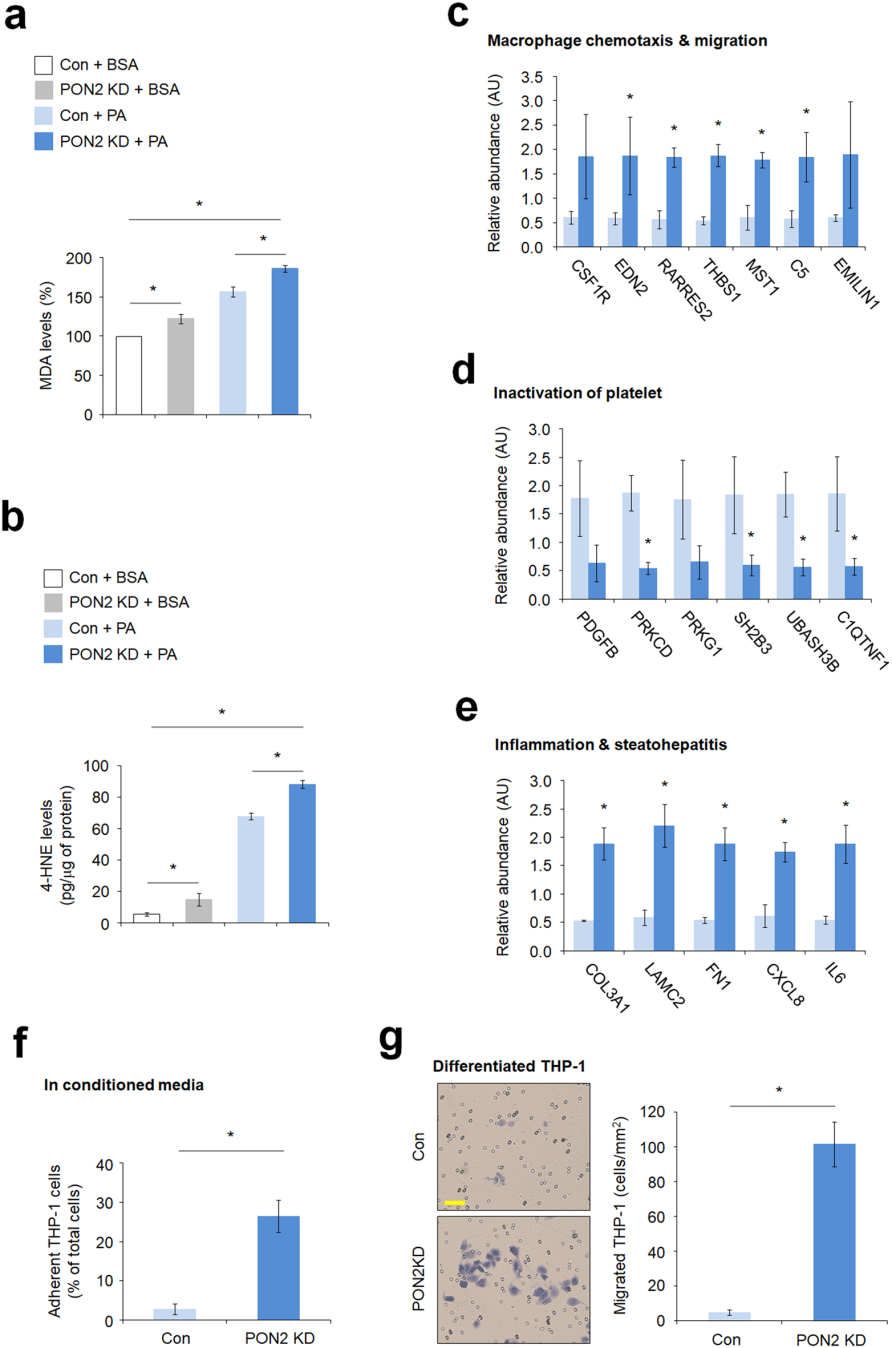
PON2 depletion increases lipid peroxidation and inflammatory response. (**a**) The quantification of the malonaldehyde adducts in PON2-deficient and control cells treated with or without PA for 24 h. Bar plots of the average lipid peroxidation. Data were obtained from three independent experiments. Error bars indicate standard deviation. (**b**) The quantification of 4-hydroxynonenal adducts in PON2-deficient and control cells treated with or without PA for 24 h. (**c**) The expression of genes related to macrophage chemotaxis and migration in PON2-deficient cells relative to that in controls. (**d**) The expression of genes related to inactivation of platelet in PON2-deficient cells relative to that in controls. (**e**) The expression of genes related to inflammation and steatohepatitis in PON2-deficient cells relative to that in control cells. (**f**) THP-1 adhesion assay in conditioned media of PON2-deficient and control cells. (**g**) Chemotaxis analysis of differentiated THP-1 macrophages against PON2 deficient and control cells. Representative images from three independent experiments were shown in the left panel (scale bar = 50 μm). Bar plots of migrated THP-1 cells/mm^2^ were shown in the right. Error bars indicate standard deviation. Statistical significance between two groups was determined with two-tailed Student’s *t* test. *p < 0.05.

Nonalcoholic steatohepatitis (NASH), an aggressive form of NAFLD, is characterized by hepatic inflammation which is linked to mitochondrial dysfunction and increased oxidative stress arising from the accumulation of oxidized mitochondrial macromolecules, specifically oxidized lipid metabolites^21,22^. Thus, we investigated the effects of PON2 depletion in the formation and accumulation of oxidized lipids. The levels of MDA adduct, an end product and the most mutagenic product of lipid peroxidation, were measured in cells. PA treatment markedly caused an increased formation of MDA adducts in control cells compared to that in cells treated with BSA (Fig. 5a), whereas PON2 depletion showed a higher accumulation of MDA adducts when compared to control cells after both BSA or PA treatment. To further validate these results, we measured the levels of 4-HNE adducts, another primary metabolites of lipid peroxidation, and similar to results of MDA adducts accumulation, PA treatment increased the levels of 4-HNE adducts in control cells when compared to that in cells treated with BSA (Fig. 5b). The levels of 4-HNE adducts were relatively higher in PON2-deficient cells than in control cells under BSA or PA treatment. These results indicate that the loss of PON2 activity causes accumulation of toxic and mutagenic lipid metabolites (4-HNE and MDA) upon excessive lipid intake, similar to that in NAFLD pathogenesis. Considering these findings, we further investigated the potential role of PON2 in the regulation of inflammatory response and analyzed the mRNA expression of genes related to the inflammatory pathway. We found that the expression levels of genes related to macrophage chemotaxis and migration, and steatohepatitis were higher in PON2-deficient cells than in control cells (Fig. 5c,e), whereas genes related to platelet inactivation were downregulated as a result of PON2 depletion (Fig. 5d). Furthermore, the monocyte activation assay showed that the adherence activity of THP-1 monocyte to the culture plate bottom was significantly higher in the PON2-KD cells than in control cells when monocytes were cultured in conditioned media (Fig. 5f). In consistent with this, macrophage chemotaxis assay showed that the migration activity of differentiated THP-1 was also significantly higher when co-cultured with PON2-KD cells than the control cells (Fig. 5g). These results indicate that the loss of PON2 activity exacerbates the inflammatory response and abnormal accumulation of oxidized lipid metabolites in hepatocytes.

### PON2 depletion decreases activation of overall autophagy pathway in hepatocytes

**Figure 6:**
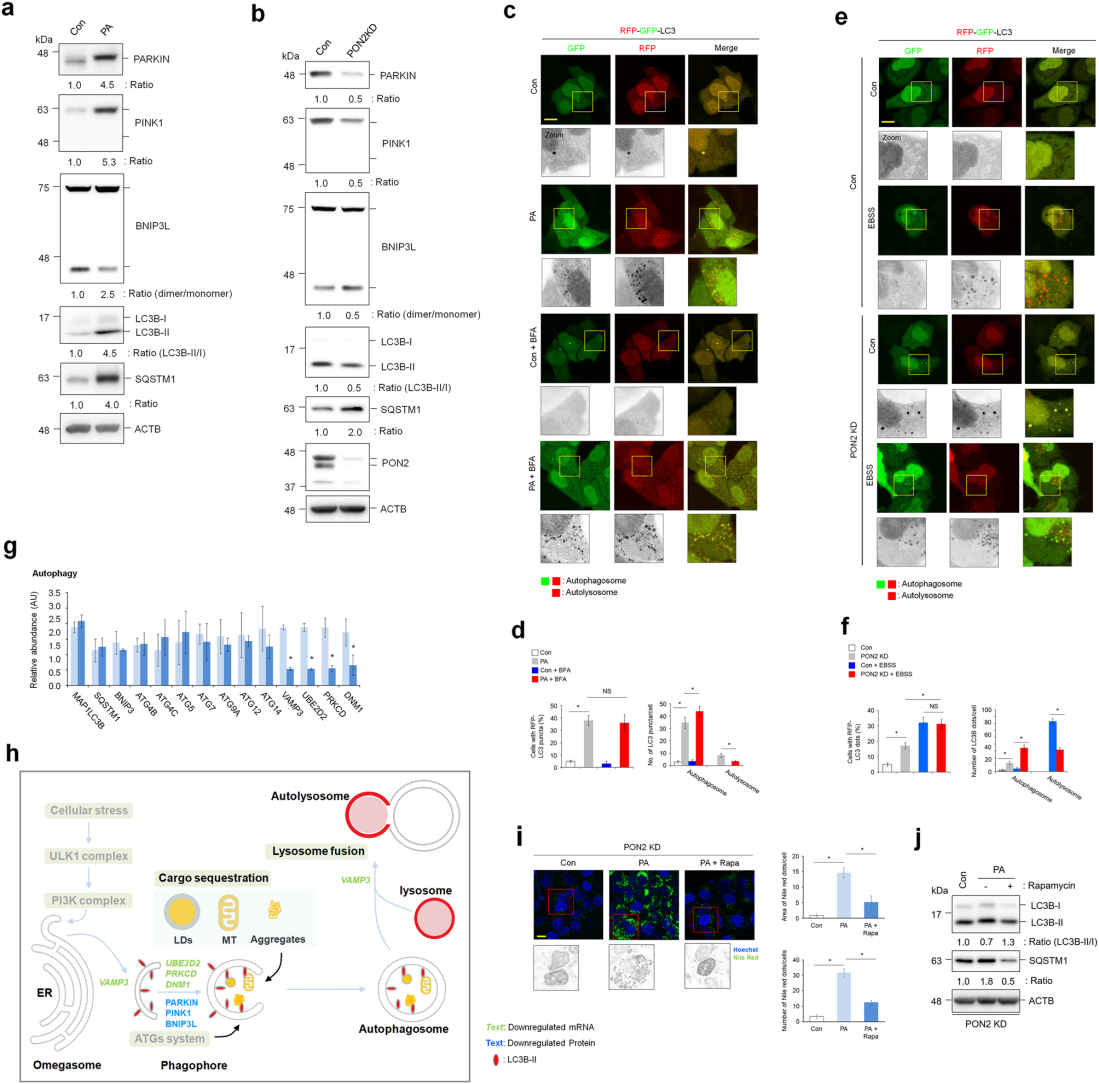
PON2 depletion inhibits autophagosome formation and autolysosome maturation. (**a**) Immunoblotting analysis of autophagy pathway activation-related markers (PARKIN, PINK1, BNIP3L, LC3B, and SQSTM1) in cells treated with PA for 24 h. ACTB was used as a loading control. The band intensities of indicated proteins are shown below. (**b**) Immunoblotting analysis of autophagy pathway activation-related markers (PARKIN, PINK1, BNIP3L, LC3B, and SQSTM1) in PON2-deficient and control cells. ACTB was used as a loading control. The band intensities of indicated proteins are shown below. (**c**) Confocal fluorescence analysis showing the PA-mediated autophagy activation. Cells were transfected with tandem fluorescent probe-tagged LC3B (red fluorescent protein (mRFP)-green fluorescent protein (GFP)-LC3B) plasmid and treated with or without PA for 24 h, followed by treatment with bafilomycin A (100 nM) for 5 h. Cells with both red and green fluorescent puncta (autophagosome) and those with only red fluorescent puncta (autolysosome) were quantified. Representative images from three independent experiments are shown above (scale bar = 50 μm). (**d**) Bar plots of the average cell numbers with red fluorescent puncta (autophagy) or average numbers of autophagosome or autolysosome per cell are shown below. (**e**) Confocal fluorescence analysis showing the autophagy activation in PON2-deficient and control cells. Cells were transfected with mRFP-GFP-LC3B plasmid, replenished with EBSS for 5 h. Cells with both red and green fluorescent puncta (autophagosome) and those with only red fluorescent puncta (autolysosome) were quantified. Representative images from three independent experiments are shown above (scale bar = 50 μm). (**f**) Bar plots of the average cell numbers with red fluorescent puncta (autophagy) or average numbers of autophagosome or autolysosome per cell are shown below. (**g**) The expression of genes related to autophagy activation in PON2-deficient cells relative to that in controls. Bar plots of the average expression of genes are shown. (**h**) Schematic representation of the hepatic autophagy pathway showing genes with significant changes in their expression in PON2-deficient cells relative to that in control cells. Downregulated mRNA and protein expression are indicated by green and blue characters, respectively. Gray characters indicate unaffected autophagy pathway in PON2-deficient cells compared to that in control cells, whereas black characters show the potentially affected pathway by PON2 depletion. (**i**) Confocal fluorescence analysis showing PA-induced lipid accumulation with or without rapamycin autophagy activator. PON2-deficient cells were treated with PA alone (100 μM) or in combination with rapamycin (100 nM) for 24 h and stained with Nile red to determine lipid accumulation. Representative images from three independent experiments were shown in the left (scale bar = 50 μm). Quantification of Nile red staining intensity which was measured as the area of Nile red dots per cell and the number of Nile red dots per cell. (**j**) Immunoblot analysis of autophagy pathway activation-related markers (LC3B and SQSTM1) in PON2-deficient cells treated with PA alone or in combination with rapamycin for 24 h. ACTB was used as a loading control. The band intensities of the indicated proteins were shown below. Error bars indicate standard deviation. Statistical significance between two groups was determined with two-tailed Student’s *t* test. *p < 0.05. NS, non-significant.

Autophagy impairment is linked to mitochondrial dysfunction and oxidative stress^23^. Defective autophagy has been directly involved in NAFLD as it plays an important role in lipid metabolism^24^. While PA treatment induces autophagy initiation, it also inhibits the activation of autophagic flux, which is linked to mitochondrial dysfunction and oxidative stress, leading to eventual lipotoxicity^25^. In the present study, immunoblot analysis revealed that the expression levels of PARKIN and PINK1, dimerization levels of BNIP3L as a marker of mitophagy activation, and the conversion level of LC3B-I to LC3B-II as a marker of autophagosome formation were increased in PA-treated hepatocytes compared to those in control cells (Fig. 6a). However, SQSTM1, whose degradation is a marker of autophagic flux, was accumulated in PA-treated cells. Therefore, similar to previous reports, our data indicate that PA stimulation promotes autophagosome formation but inhibits autophagic flux in hepatocytes. Defective PON2 activity also decreased the expression of PARKIN and PINK1, dimerization levels of BNIP3L, and the conversion levels of LC3B-I to LC3B-II (Fig. 6b). However, the level of SQSTM1 increased in PON2-deficient cells compared to that in control cells. Unlike the results of PA treatment, these results indicate that PON2 depletion decreases both autophagosome formation and autophagic flux. Autophagy activation by PA stimulation or PON2 depletion were verified using the autophagy indicator tandem fluorescence-tagged LC3B. The number of autophagosomes in PA-treated cells was markedly higher than that in the control cells, as evidenced by the accumulation of both RFP and GFP puncta (Fig. 6c,d). When the lysosomal fusion inhibitor bafilomycin A was added to cells, the accumulation of autophagosomes in PA-treated cells decreased, indicating that PA induces autophagosome formation, but inhibits autophagic flux. Similar to the results of the PA treatment, the number of cells with autophagosomes were higher in the PON2-deficient cells than in the control cells (Fig. 6e,f). The effect of PON2 on autophagic flux was closely examined by autophagy induction via starvation with EBSS. Upon EBSS treatment, the number of autolysosome (RFP puncta) in control cells markedly increased, but the number of autophagosome did not increase, relative to that of normal culture media, indicating an activation of autophagic flux by EBSS in the control cells. However, the number of autolysosomes in PON2-deficient cells was marginally increased compared to that in control cells treated with EBSS. These results indicate that PON2 depletion inhibits autophagic flux, similar to PA treatment.

To investigate the mechanisms underlying PON2-mediated autophagy activation, the mRNA expression levels of genes related to regulation of autophagy pathways were analyzed. Among genes related to regulation of autophagy, PON2 depletion remarkably decreased expression of genes involved in phagophore maturation, lysosome fusion (*VAMP3*), and cargo sequestration (*UBE2D2, PRKCD, and DNM1*), but did not affect the expression of genes related to the autophagy system (*ATG4C, ATG5, ATG7, ATG9A, ATG12, and ATG14*) and autophagy initiation (*SQSTM1 and MAP1LC3B*; Fig. 6g). Taken together, our data indicate the potential role of PON2 in regulation of overall autophagy pathways (Fig. 6h). Notably, PON2 was found to be associated with the regulation of mitophagy activation as evidenced by the changes in the expression of mitochondrial cargo receptors (*PRKCD* and *DNM1* mRNAs; PARKIN, PINK1, and BNIP3L proteins). Lastly, to show the association between lipid accumulation and autophagy activity in PON2-deficient cells, we analyzed lipid accumulation after treatment with the autophagy inducer rapamycin. Rapamycin treatment significantly reduced the PA-mediated lipid accumulation (Fig. 6i) and induced autophagy activation in PON2-KD cells (Fig. 6j). These data indicate that PON2-mediated regulation of autophagy activation is important for the lipid catabolism in hepatocytes.

## Discussion

Despite the clinical importance of NAFLD as one of the most common chronic liver diseases worldwide, the study for the drug target for effective NAFLD treatment has not been entirely satisfactory. Therefore, an effective treatment for NAFLD is still absent. Although the pathogenesis of this disease is associated with multifactorial and complex parameters, it has been well-known that mitochondrial dysfunction and autophagy impairment are some of the major causative factors in NAFLD pathogenesis^24,26–28^. In agreement with a previous report on HFD-induced NAFLD animal model^14^, we demonstrated here that PON2 activity is inhibited in a PA-induced in vitro fatty liver model, whereas its expression level is not affected. We found that PON2 depletion could disrupt and abnormally modulate the activation of numerous functional pathways that are closely related to NAFLD pathogenesis. The PON2-deficient hepatocytes resembled many features present in the liver of patients with NAFLD, especially the mitochondrial dysfunction, excessive oxidative stress, and increased lipid accumulation. We demonstrated that PON2 depletion is directly associated with the pathogenesis of steatohepatitis and liver fibrosis, as evidenced by the mRNA expression of genes related to the regulation of macrophage activation, collagen metabolism, and other inflammation-related pathways. Our in vitro phenotypic results showed that PON2 contributes to the hepatocyte protection and attenuation of macrophage chemotaxis to hepatocytes under lipotoxic condition. These data indicate a potential role of PON2 that may contribute to the regulation of autophagy pathways, including phagophore maturation, autophagic cargo sequestration, and autolysosome maturation processes. Indeed, PON2-mediated autophagy activation is closely related to the alleviation of the lipid accumulation in hepatocytes. These findings reveal a critical role of PON2 in NAFLD pathogenesis and validate its potential as a candidate gene for the development of effective drugs for NAFLD treatment.

Fundamentally, LDs are dynamic intracellular lipid storage organelles that respond to the physiological state of cells, facilitate lipid transfer into mitochondria, and control metabolism^28,29^. However, excessive accumulation of LDs observed in hepatocytes of patients with NAFLD results from disruption in lipid metabolism^30^. In the present study, PON2 depletion in hepatocytes showed increased accumulation of LDs through inhibition of lipolysis. Our transcriptomics approach facilitated the understanding of the regulatory mechanism behind PON2-associated biological processes. Among the various biological processes, lipid metabolism was modulated by PON2 depletion, where the hepatic expression of genes associated with cholesterol and ceramide metabolism were unregulated in PON2-deficient cells, whereas the expression of genes related to FA metabolism was downregulated. The accumulation of cholesterol as well as triacylglycerol because of disturbance of lipid metabolism in hepatocytes, is closely associated with NAFLD pathogenesis^31^. Thus, our data strongly suggest that the loss of PON2 activity in hepatocytes is associated with stimulation of de novo lipogenesis and disruption of lipolysis activity, which may lead to the exacerbation of fat accumulation in the liver of patient with NAFLD, who are predisposed to a decreased capacity of FA utilization. Ceramide, a class of sphingolipids, causes liver tissue damage, and its increased synthesis is considered one of the critical factors in the development and progression of NAFLD^32^. Thus, we suggest that PON2 deficiency in hepatocytes may cause liver damage, through the increased ceramide synthesis, consequentially leading to severe progression of NAFLD to steatohepatitis and liver fibrosis.

Unlike PON1 and PON3 that are primarily secreted and functional in serum, PON2 is localized to intracellular regions, especially in the mitochondria and the endoplasmic reticulum^12^. Although PON2 acts as an intracellular antioxidant protein, the molecular mechanism of how PON2 reduces oxidative stress has not been fully elucidated. PON2 deficiency in mice leads to decreased mitochondrial ATP levels, accompanied by increased mitochondrial oxidative stress^33^. In in vitro fatty liver model, our data also reveal that PON2 depletion in hepatocytes decreases mitochondrial respiratory capacity and increases ROS production. Unlike glycolysis, excessive FA catabolism increases ROS production in the mitochondria^18^, which leads to oxidative stress and lipotoxicity. In the present study, PON2 depletion decreases FA oxidation, while simultaneously increasing mitochondrial ROS production and JNK1/2 activation, which are indicators of oxidative stress. Mechanistically, the disruption of mitochondrial ETC complex causes an increase in ROS production^34^. Moreover, the impairment of mitochondrial respiratory chain indirectly induces oxidative stress through the ER^35^. Our data show that PON2 depletion downregulates the genes related to the mitochondrial ETC and TCA cycles. Thus, the increased mitochondrial oxidative stress in PON2-deficient cells may be closely associated with the reduced activity of mitochondrial ETC complex. These findings suggest that PON2 contributes to mitochondrial metabolism and antioxidant functions through the transcriptional regulation of genes related to mitochondrial respiratory capacity, unlike secreted PON1 that acts as direct antioxidants via its own enzymatic process^36^.

All members of the PON family have been implicated in the pathogenesis of inflammatory diseases such as atherosclerosis^37^, although the underlying mechanism of how PON2 is involved in inflammatory response of NAFLD has not been reported. Generally, oxidative stress not only disrupts hepatic FA oxidation but also induces liver inflammation via the production of oxidized lipid metabolites^34,38^. We demonstrated that PON2 depletion in hepatocytes could increase oxidative stress and lipid accumulation by dysregulating mitochondrial systems. Our data also reveal that PON2 deficiency increases lipid peroxidation in hepatocytes, under both normal and PA treatment, as evidenced by the accumulation of MDA and 4-HNE adducts. Corresponding to the increase in lipid peroxidation, PON2 deficiency triggered an inflammatory response in hepatocytes, as evidenced by the upregulation of genes related to macrophage infiltration and pro-inflammatory cytokines. Therefore, our data suggest that the loss of PON2 activity leads to the hepatic inflammation owing to increased lipid peroxidation.

Autophagy is a lysosomal degradation pathway that maintains cellular homeostasis by supplying energy^39^. Particularly, autophagy is also involved with the breakdown of intracellular lipids to enhance lipid catabolism in hepatocytes, and impaired autophagy is highly relevant to the pathogenesis of NAFLD^9^. PA reduces autophagic flux, which is involved in the hepatic lipotoxicity^25,40^. Furthermore, the pharmacological activation of autophagy attenuates PA-induced oxidative stress and prevents hepatocytes from lipotoxicity^41,42^. In the present study, we showed that PON2 depletion reduces overall efficiency of the autophagy pathway. Together with the reduced expression of genes related to lipid metabolism in PON2-deficient cells, our data suggest that autophagy inhibition by PON2 deficiency may impair lipid catabolism because of deficient FA supply from LDs to mitochondria.

Mitophagy is a form of autophagy that selectively degrades damaged mitochondria, following stress conditions, such as excessive lipid catabolism in the liver of patient with NAFLD^8,43^. The interplay between mitophagy and mitochondrial biogenesis promotes mitochondrial recycling, which contributes markedly in maintaining mitochondrial and cellular homeostasis upon normal and metabolic stress conditions^8,43^. Indeed, mitophagy reduces ROS accumulation by clearing damaged mitochondria, leading to cellular protection against oxidative stress^44^. The damaged mitochondria are selectively removed through recognition via the mitochondrial cargo receptor and subsequent lysosomal degradation^43,45^. Our study shows that PON2 depletion decreased the expression of the *VAMP3* gene, which is associated with phagophore maturation and lysosome fusion^46^, suggesting that PON2 is involved in the autophagic flux dynamics, as well as autophagosome formation. Importantly, our data also show that PON2 depletion could induce the downregulation of not only PARKIN, PINK1, and BNIP3L protein but also *PRKCD* and *DNM1* mRNAs, both of which are mitophagy-specific cargo receptors^45,47,48^. These results demonstrate that PON2 is specifically related to the regulation of mitochondrial cargo sequestration and, therefore, one may hypothesize that PON2 acts on the mitophagy-mediated pathway to alleviate cellular oxidative stress.

PON2 is a multifunctional protein that acts both enzymatically and non-enzymatically and has been implicated in the pathology of various diseases. The enzymatic activity of PON2 is involved in the regulation of inflammatory responses and defense against pathogens^12,49^. PON2 hydrolyzes polyunsaturated FAs such as arachidonic acid and docosahexaenoic acid and synthesizes the prostaglandin E2 (PGE2), inhibiting the macrophage activation and attenuating the inflammatory response^49^. Indeed, PON2 inhibits *N*-acylhomoserine lactones through enzymatic hydrolysis^12^, thereby interfering with the quorum sensing in pathogenic bacteria. The physiological significance of PON2 enzymatic activity represents the regulation of inflammatory diseases, which suggests that the loss of PON2 enzymatic activity may be a major cause of exacerbating inflammation in NAFLD patients. In addition to enzymatic activity, PON2 also acts non-enzymatically. PON2 is localized in the plasma membrane and protects membrane lipids from non-enzymatic peroxidation^50^. Lipid peroxidation products such as 4-HNE activate autophagy but inhibit autophagic flux^51^. In our study, PON2 deficiency in hepatocytes leads to the accumulation of lipid peroxidation products and inhibition of autophagic flux under lipotoxic conditions. Thus, accumulation of 4-HNE in PON2-deficient cells may be associated with the inhibition of autophagic flux in hepatocytes. Indeed, the non-enzymatic activity of PON2 can modulate metabolism through physiological interaction with GLUT1^52^. Taken together, loss of PON2 function is associated with the pathology of metabolic liver diseases such as NAFLD, but it is unclear whether loss of enzymatic activity of PON2 under lipotoxic conditions affects its non-enzymatic activity through the conformational modification.

In conclusion, this study investigated an in vitro fatty liver model to demonstrate that PON2 functions by regulating various key features of NAFLD, including mitochondrial homeostasis, lipid metabolism, and inflammation. Notably, we found a new role for PON2 in regulating the overall autophagy pathway, which is its underlying mechanism, and holistically attenuating the features of NAFLD. Our findings contribute to the understanding of the cellular basis of NAFLD and propose PON2 as a novel target protein for the development of therapeutics for NAFLD.

## Methods

### Reagents and antibodies

Bovine serum albumin (BSA; A1595), palmitic acid (PA; P0500), Nile red (19123), p-nitrophenyl acetate (pNPA; N8130), 5′,5-dithiobis (2-nitrobenzoic acid) (DTNB; D8130), MTT cell growth assay kit (CT02), phorbol 12-myristate 13-acetate (PMA; P8139), and Rapamycin (R0395) were purchased from Sigma Aldrich (St. Louis, MO). Furthermore, 4% paraformaldehyde (BP031a) was purchased from Biosolution (Seoul, Republic of Korea). MitoSOX red mitochondrial superoxide indicator (M36008), Hoechst 33342 (H1399), and protease inhibitor cocktail (PIC; 78441) were purchased from Thermo Fisher Scientific (Waltham, MA, USA). For immunoblotting, the following antibodies were used: anti-PON2 (Abcam, ab183718), anti-ACTB (Sigma, A5441), anti-phospho-c-Jun N-terminal kinase (JNK) 1/2 (Cell Signaling Technology, 9251), anti-JNK1/2 (Cell Signaling technology, 9252), anti-PARKIN (Cell Signaling Technology, 2132), anti-PINK1 (Novus, BC100-494), anti-BNIP3L (Cell Signaling Technology, 12396), anti-LC3B (Cell Signaling Technology, 2775), anti-SQSTM1 (Cell Signaling Technology, 5114), anti-mouse conjugated with horseradish peroxidase (Cell Signaling Technology, 7076), and anti-rabbit conjugated with horseradish peroxidase (Cell Signaling Technology, 7074).

### Cell culture and generation of stable *Pon2* knockdown cells

L02 cells (an immortalized normal liver cell line) were a kind gift from Dr. KH Lee (Korea Institute of Radiological and Medical Sciences). THP-1 cells (human acute monocytic leukemia cell line) were purchased from Korean Cell Line Bank (KCLB NO. 40202). The L02 cells were cultured in Dulbecco’s modified Eagle’s medium (DMEM; LM001-05, Welgene), and THP-1 cells were cultured in RPMI 1640 medium (SH30027.01, Cytiva) supplemented with 10% fetal bovine serum and 100 U/mL of penicillin and streptomycin at 37 °C in a 5% CO_2_ incubator.

Stable PON2 knock-down (KD) L02 cells were generated by transducing the cells with recombinant lentivirus harboring short hairpin RNA against PON2 (shPON2; Gencopoeia, LPP-HSH013480-LVRU6P). The resultant PON2-KD cells were cultured in DMEM supplemented with puromycin (500 ng/mL; A11138-03, Life Technologies). The PON2 KD was confirmed using immunoblotting with the anti-PON2 antibody.

To prepare the BSA-conjugated PA solution, 100 mM PA solution was prepared in 0.1 mM NaOH and the solution was heated at 70 °C. PA solution was incubated with 10% BSA at 55 °C for 30 min to obtain 5 mM PA/1% BSA^53^. The solution was then cooled to 25 °C, filter-sterilized, and stored at −20 °C until use. The cells were incubated with culture medium containing 100 µM BSA-conjugated PA or BSA as control treatment.

### Lipid accumulation assay

Lipid accumulation assay was performed as described previously^54^. L02 cells were plated in six-well plates and incubated with 100 µM PA for the indicated durations. Next, the cells were fixed with 4% paraformaldehyde for 10 min and were stained with 1 µg/ml Nile red solution for 15 min at room temperature. After one wash with PBS, the images were acquired using a Carl Zeiss Confocal LSM710 Meta microscope (Carl Zeiss, Seoul, Republic of Korea). The fluorescence intensity was quantified using the National Institutes of Health (NIH) ImageJ software.

### Cell viability assay

Cells were plated in 12-well plates at an initial density of 5 × 10^5^/well and treated with 250 and 500 µM PA for 24 h. MTT solution was added to each well to a final concentration of 0.5 mg/mL. After exposure at 37 °C for 4 h, formazan crystals were dissolved in DMSO. Absorbance was measured at 570 nm.

### Detection of mitochondrial superoxide

Mitochondrial superoxide was measured as described previously^55^. Cells were plated in six-well plates and incubated with 100 µM PA for 24 h. Next, the cells were pulsed with 2.5 µM MitoSOX red mitochondrial superoxide indicator and 5 µM Hoechst 33342 for 30 min. After washing, the cells were observed under a Carl Zeiss Confocal LSM710 Meta microscope in a chamber heated to 37 °C at 5% CO_2_. The fluorescence intensity was quantified using the NIH ImageJ software.

### Immunoblotting analysis

To extract proteins, the cultured cells were lysed using sodium dodecyl sulphate (SDS) lysis buffer (100 mM Tris–HCl, pH 6.8, 10% glycerol, and 1% SDS), supplemented with PIC^56^. The protein concentration was determined using the BCA protein assay kit (23225, Thermo Fisher Scientific). The samples were boiled in 1 × sample buffer (10 mM Tris–HCl, pH 6.8, 1% SDS, 5% glycerol, 0.05% bromophenol blue, and 1% β-mercaptoethanol) for 5 min and then subjected to SDS–polyacrylamide gel electrophoresis. The resolved proteins were electro-transferred onto an Immobilon-P membrane (Merck, IPVH00010). Finally, the membrane was probed with specific antibodies; thereafter, immunoreactive signals were detected using a LAS-4000 Luminescent Image Analyzer (GE Healthcare Bio-Sciences). The signal intensity was assessed by measuring the relative density of each band and normalizing it to that of ACTB using the Multi Gauge software (Fujifilm).

### Quantification of PON2 enzyme activity

#### Endogenous PON2 activity

Cells were plated into six-well plates and incubated with 100 µM PA for 24 h; subsequently, the cells were scrapped and centrifuged to collect cell pellets that were washed with PBS and stored at − 80 °C until use. The frozen cell pellets were incubated with 25 mM Tris buffer (pH 7.4) containing 0.05% n-dodecyl-β-D-maltoside (D4641, Merck) and 1 mM CaCl_2_ and lysed by subjecting them to three freeze–thaw cycles.

For evaluating the PON2 esterase activity^57^, pNPA hydrolysis was determined using a SpectraMax Plus384 microplate reader (Agilent Technologies, Seoul, Republic of Korea). The cell lysates were transferred to a 96-well plate, and reactions (0.2 mL final mixture volume) were initiated by adding 1 mM pNPA in PON2 activity assay buffer (50 mM Tris, pH 8.0, and 1 mM CaCl_2_). Absorbance at 412 nm, resulting from the release of p-nitrophenol, was recorded.

For evaluating the PON2 lactonase activity^58^, the enzymatic hydrolysis of the thioalkyl-substituted lactones was determined. The cell lysates were transferred to a 96-well plate, and reactions were initiated by adding 1 mM 5-thiobutyl butyrolactone (TBBL) and 1 mM DTNB in PON2 activity assay buffer. The enzymatic hydrolysis was monitored by examining the absorbance of the reaction mixture at 420 nm.

#### Recombinant PON2 activity

For performing the oxidized linoleic acid (Ox-LA)-mediated PON2 inhibition assay^59^, purified recombinant PON2 protein (10 µM) was incubated with or without 100 µM Ox-LA in the PON2 activity assay buffer in a 96-well plate for 10 min at room temperature. To evaluate the esterase activity, the reactions were initiated by adding 1 mM pNPA in PON2 activity assay buffer. The enzymatic hydrolysis was monitored by measuring the absorbance of the reaction mixture at 412 nm. To evaluate the lactonase activity, the reactions were initiated by adding 1 mM TBBL and 1 mM DTNB in PON2 activity assay buffer. The enzymatic hydrolysis was monitored by measuring the absorbance of the reaction mixture at 420 nm.

### RNA-sequencing (RNA-seq) and data analyses

Qiagen RNeasy Kit (Qiagen, Hilden, Germany) was used for cell lysis and RNA purification of both L02 liver cells and L02 PON2-KD cells. RNA quality was assessed using an Agilent Bioanalyzer, and the samples had average RNA Integrity Number (RIN) value of 8.1 ± 0.7 and 7.3 ± 0.9, respectively. The libraries were prepared with the Illumina TruSeq Stranded mRNA kit and sequenced using NovaSeq 6000 platform (Illumina, San Diego, CA, USA) yielding 101-bp paired end reads. After quality control and alignment to the human reference genome using HISAT2 program for read mapping, transcript assembly was performed with the StringTie program. Differentially expressed genes were identified using DESeq2.

Z-score-based analysis was performed to identify differentially expressed genes (DEGs) in which genes with significant z scores (cutoff value = 1.20) were selected using the Perseus software (v.1.6.1.1). A GO search was performed using g-Profiler to explore pathways in GO-BP, Reactome, and KEGG. GO-BPs with a *p* value < 0.05 were identified as enriched by DEGs. To construct a network depicting enriched processes, GO-BP-based network analysis was visualized and interpreted in Cytoscape using Enrichment Map.

### Measurement of oxygen consumption rate (OCR)

OCR was measured using XFp Extracellular Flux Analyzers (Agilent Seahorse Biosciences). The cells were plated into XFp cell culture mini plates for 24 h, and the culture medium was replaced with XF-Base medium (non-buffered Roswell Park Memorial Institute-1640 medium containing 2 mM L-glutamine, 1 mM sodium pyruvate, and 10 mM glucose, pH 7.4) for 30 min. Next, the cells were incubated with PA-BSA or BSA control (Seahorse XF PA-BSA FAO Substrate, Agilent Seahorse Biosciences, 102720-100) and analysis was performed using the XF assay with Seahorse XF Long Chain Fatty Acid Oxidation Stress Test kit (Agilent Seahorse Biosciences, 103672-100). Three measurements were recorded under basal conditions and after the addition of 2 µM oligomycin, 0.5 µM carbonyl cyanide-p-trifluoromethoxyphenylhydrazon (FCCP), and 1 µM rotenone/antimycin. OCR values were normalized to the cell number.

### Lipid peroxidation assay

Lipid peroxidation in the cells was determined by measuring the levels of malondialdehyde (MDA), by indirectly measuring the formation of TBARS, which is a chromogen derived from the reaction of MDA with the thiobarbituric acid reaction. The cells were plated in six-well plates and incubated with 100 µM PA for 24 h. Next, the cells were trypsinized and centrifuged; then, the cell pellets were washed with PBS and stored at − 80 °C until use. The MDA concentration was determined using the TBARS parameter assay kit (R&D systems, KGE013) following the manufacturer’s instructions.

For performing the 4-hydroxynonenal (4-HNE) assay, the cells were lysed using RIPA buffer (Cell Signaling Technology) with PIC. The protein concentration was determined using the BCA protein assay kit. The 4-HNE concentration was determined using the 4-HNE ELISA kit (BioVision, E4645-100) following the manufacturer’s instructions.

### Autophagy flux analysis

Immunoblotting analysis was performed to analyze the endogenous LC3B-II/I and SQSTM1/p62 expression levels with anti-LC3B and anti-SQSTM1 antibodies, respectively, following the manufacturer’s instructions. The cells were plated in six-well plates and incubated with 100 µM PA for the indicated durations. Next, the cells were lysed with SDS lysis buffer with PIC. The proteins were subjected to immunoblotting, and the intensity of each protein signal was normalized to that of ACTB. The control value was set to 1.0 and the protein intensity was represented, relative to that of the control.

To monitor the autophagy level^60^, microtubule-associated protein 1A/1B-light chain 3(LC3B) puncta in cells which were transfected with mRFP-GFP tandem fluorescent-tagged LC3B (tfLC3B) were examined using fluorescence microscopy. The cells cultured on glass coverslips were transfected with mRFP-GFP tfLC3B plasmid. At 16 h post-transfection, the cells were treated with 100 µM PA in the presence or absence of bafilomycin A1 (Invivogen, tlrl-baf1). PON2-deficient cells were treated with or without EBSS for inducing starvation condition. Cellular localization of LC3B was observed using a Carl Zeiss Confocal LSM710 Meta microscope, and the images were processed with the software supplied by the manufacturer and analyzed with NIH ImageJ software. Cells containing three or more mRFP-LC3B puncta were defined as autophagy-positive cells. The percentage of autophagy-positive cells relative to the total number of mRFP-positive cells was calculated. At least 100 mRFP-positive cells per sample were counted in at least three different independent experiments. Cells stained with both red fluorescent protein (RFP) and green fluorescent protein (GFP) were defined as autophagosome-positive, whereas those stained with only RFP were defined as autolysosome-positive. The number of fluorescent LC3B puncta was determined by counting more than 100 cells with triplicates.

### Monocyte adherence and macrophage chemotaxis assay

Assay of monocyte adhesion to plastic surface was performed using the conditioned media of L02 cells as previously reported^61^. THP-1 monocytes were seeded in 6-well plates at 5 × 10^5^/mL and stimulated with conditioned media of either PON2-KD or Con L02 cells for 24 h. THP-1 cells adhering to the bottom surface of the plates were removed with cold 0.5 mM PBS-EDTA and counted. Non-adherent cells in supernatant were also harvested and counted. Adhesion rate refers to the ratio of adherent cells to the total number of cells (adherent + non-adherent).

Macrophage chemotaxis assay was performed using 6-well transwell inserts (8 μm pore size membrane, BD Biosciences) as previously reported^62^. To generate THP-1-derived macrophages, 1 × 10^6^ THP-1 cells were treated with 100 ng/mL PMA for 48 h and adherent cells were collected. THP-1-derived macrophages (5 × 10^5^ cells/well) were placed in the upper chamber of the transwell inserts with 1 mL of serum-free medium. PON2-KD or Con cells in 2 mL DMEM containing 10% FBS were added in the lower chambers of the transwell plate. Cells were incubated at 37 °C in a 5% CO_2_ incubator for 48 h. Cells that migrated down the membrane were fixed with 4% paraformaldehyde and stained with 0.4% trypan blue. Five random fields were photographed and counted from each well. Migrated cells represent the number of cells per mm^2^.

### Statistical analyses

All data, except RNA-seq data, were analyzed using GraphPad Prism v5.02 software (GraphPad, La Jolla, CA). All experiments were repeated at least three times. Data are presented as the mean ± standard error, unless specified otherwise. Data between two groups were analyzed using the Student’s *t*-test and multi-group comparisons were performed using one-way analysis of variance (ANOVA). Differences were considered significant at *p* < 0.05.

## Supplementary Information

Supplementary Figures.

## Data Availability

All data supporting the findings of this study are available within the article and its Supplementary Information files and from the corresponding author upon reasonable request. RNA-Seq datasets used in this study is publicly available and can be found in the GEO database (https://www.ncbi.nlm.nih.gov/geo/) (accession number: GSE213653).
