# Supplementary material for: Paraoxonase-2 contributes to promoting lipid metabolism and mitochondrial function via autophagy activation
Source: Sci Rep. 2022 Dec 12;12:21483. doi: 10.1038/s41598-022-25802-1 (PMC9744871; doi:10.1038/s41598-022-25802-1)
Supplement: Supplementary file 1 — Supplementary Figures. [file 41598_2022_25802_MOESM1_ESM.docx]

**Supplemental information**

Paraoxonase-2 contributes to promoting lipid metabolism and mitochondrial function via autophagy activation

Gu-Choul Shin, Hyeong Min Lee, Na Yeon Kim, Sang-Ku Yoo, Hyung Soon Park, Leo Sungwong Choi, Kwang Pyo Kim, Ah-Ra Lee, Sang-Uk Seo, and Kyun-Hwan Kim


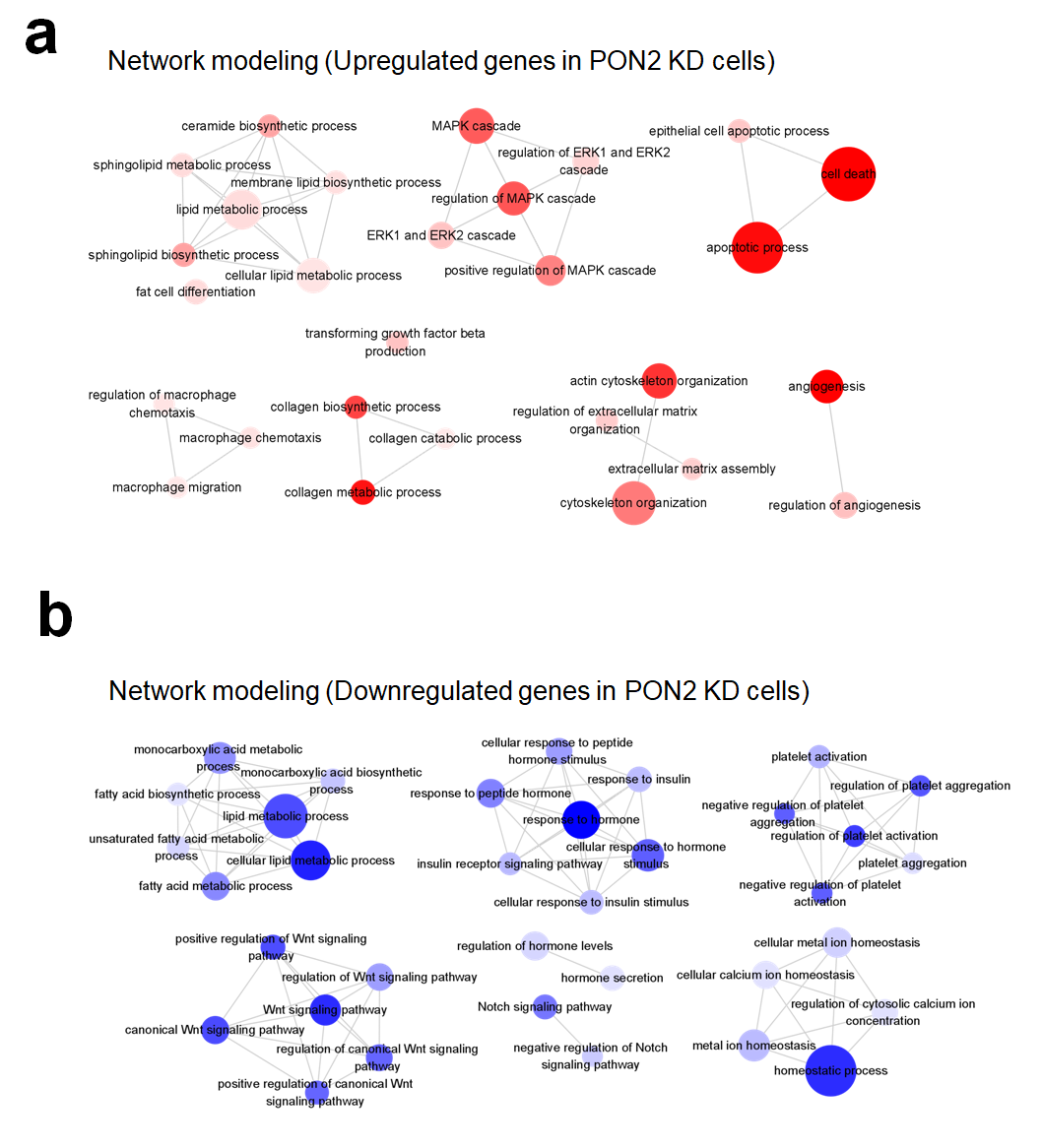


**Supplementary Fig. 1. Reactome functional interaction network and their interactions of differentially expressed genes in PON2-deficient and control cells.** **a** Network modeling of upregulated genes in PON2-deficient cells relative to that in control cells. **b** Network modeling of downregulated genes in PON2-deficient cells relative to that in control cells. Circle size represents the gene count of each process, with the circle color proportional to module size –Log (P-value).


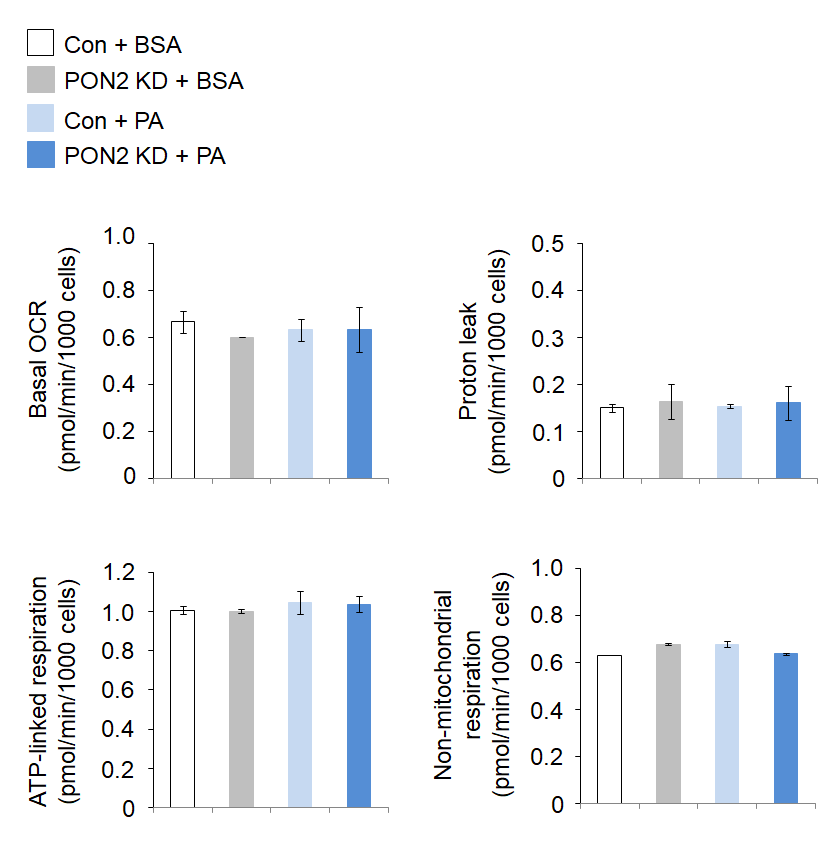


**Supplementary Fig. 2. Analysis of oxygen consumption rate (OCR) in PON2-deficient cells and control cells treated with or without PA.** Bar plots of basal OCR, ATP-linked respiration, proton leak, and non-mitochondrial respiration are shown. Data were obtained from three independent experiments. Error bars indicate standard deviation.


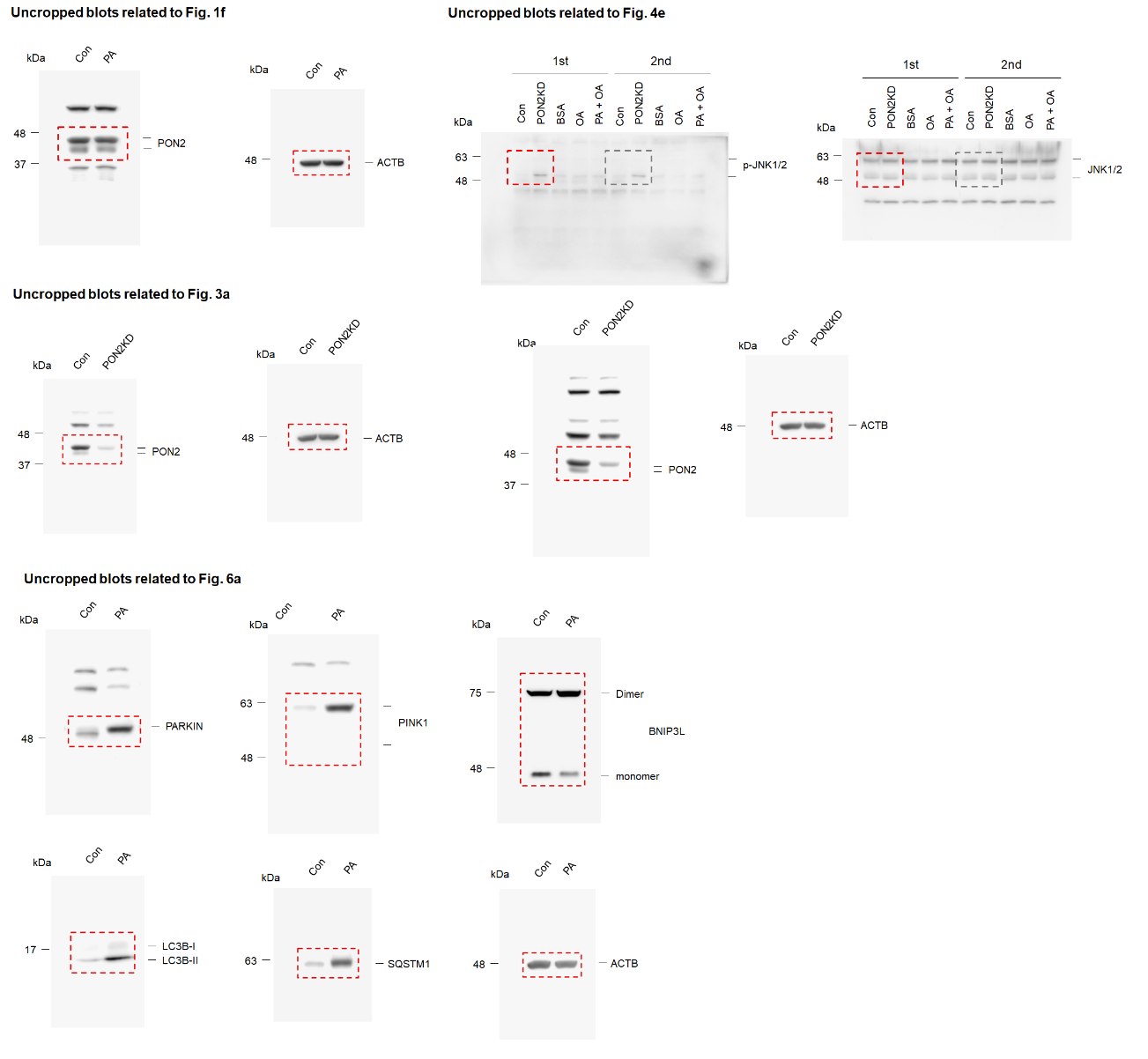


**Supplementary Fig. 3. Unprocessed data for immunoblots**


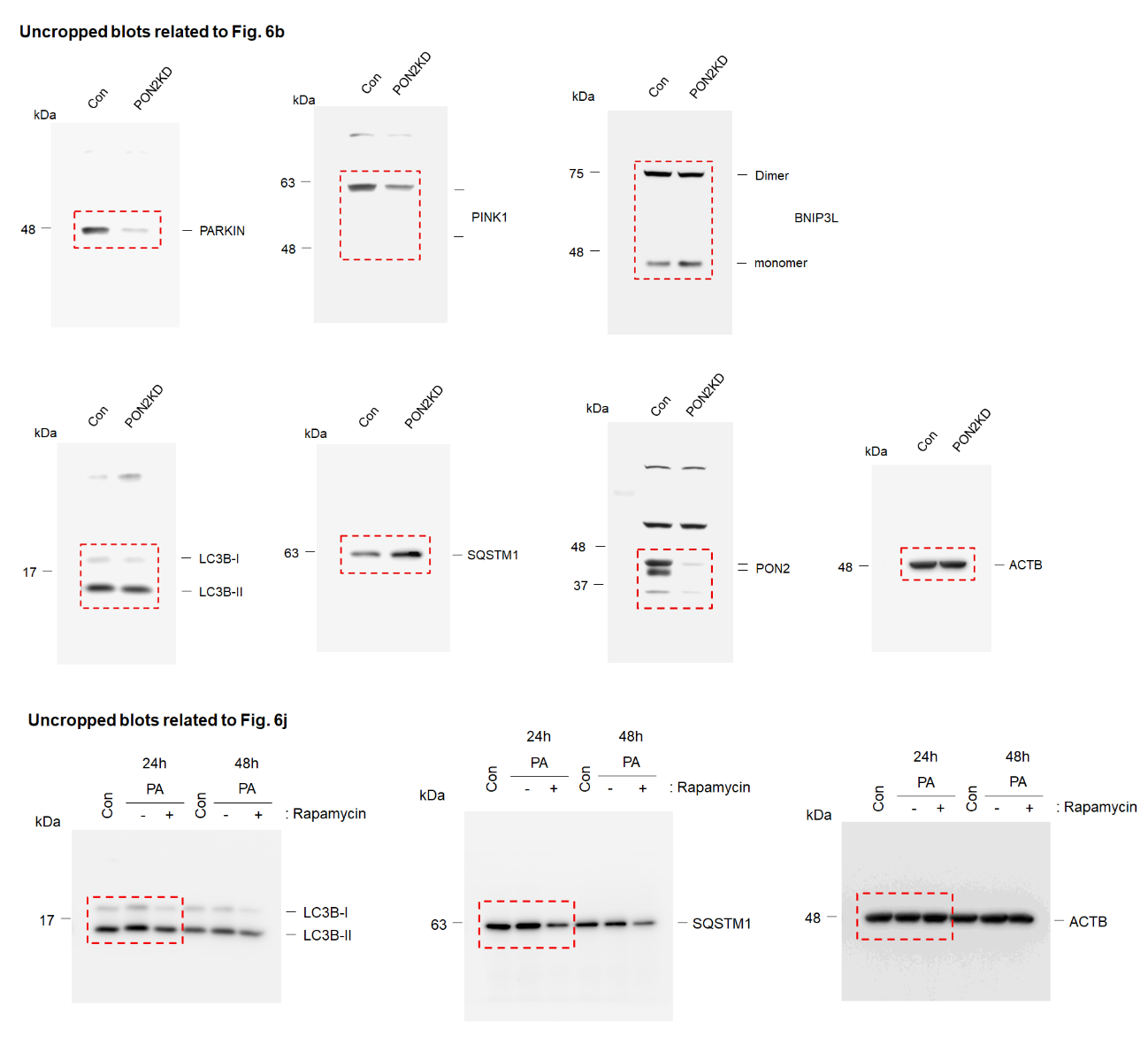


**Supplementary Fig. 3. Continued**
